# Illinois State Board of Health

**Published:** 1886-01

**Authors:** 


### STATE BOARDS OF HEALTH.

---

THE ILLINOIS STATE BOARD OF HEALTH.

The quarterly meeting of *The Illinois State Board of Health* was held at Springfield on October 29 and 30.

The Secretary's report showed that 107 certificates for practice had been issued in the quarter ending September 30, upon diplomas of colleges in good standing.

Three were granted for a certain number of years' practice in the State, and seven duplicates were supplied for lost originals.

Thirty-four applications were refused, owing to the applicants' inability to reach the required standard in examination.

The Board has now ready for publication a Conspectus of Colleges, which will be embodied in the seventh annual report. This valuable conspectus will show the comparative status of every medical college as to requirements, curricula, fees, etc. The effect of this publication and of those which have preceded it will be seen in the alleged number of irregularities, evasions of requirements, granting of diplomas in violation of published and usual conditions—charges which are constantly being presented to the board.

A graduate of a school that has repeatedly found fault with the Board, charging that its graduates were not treated fairly, and heretofore failed to announce the usual preliminary education requirements before admitting students to its classes, although at the same time claiming that the requirements were fully carried out, applied during the quarter for a certificate based upon a diploma issued June 2, 1885. In accordance with the rule of the Board exacting an examination in branches or subjects of the Schedule of Minimum Requirements omitted or neglected by the college from which the applicant is graduated, the certificate was withheld pending such examination. The matter terminated by the withdrawal of the application, the applicant having "a chance to do better" in a neighboring State. His fitness for the study and the practice of medicine, and the manner in which the school enforces the preliminary-education requirement, may be inferred from this final letter:

——— Ill July 1t '85

Mr J H Rauch Sir

In Reply woul Say that I red one year With ——— & with ——— of Ohio 3 years Practiced 3 years tend Lectures the Winter of 80 & 81 Practiced in ——— CO Ohio up to December last Then was at College untel the close on June 2th 85 Was born in ——— CO Ohio 1837 Taught there & in this State up to the time of Reading Med thin Dropeted off I was Requiered to furnish a Certificate on My first Course of Lectures Also a Recommendation from My Precepter I have fulfilled the Requierements of the Collage and Came here to practice but have a Chance to do better away from here
—have Baught an Intrust in a Drug Store in Ind you will send my Diploma to ——— Ill if you will send a Certificate I will be Obliged to you if not all Rite as I am going out of the State at all evence My age is 48 years Tought 7 Schools 5 Winters in Ohio (& 2 in this State in 1864 & 5) now it Remains with you the State Board Act on yours as Ever Truley

